# Manual of Dental Surgery and Pathology

**Published:** 1882-06

**Authors:** Alfred Colemn


### MANUAL OF DENTAL SURGERY AND PATHOLOGY.

BY ALFRED COLEMN, L.R.C.P., F.R.C.S., ETC.

Senior Dental Surgeon, and Lecturer on Dental Surgery to St. Bartholomew's Hospital; Senior Dental Surgeon and Lecturer on Dental Surgery to the Dental Hospital of London; Member of Board of Examiners in Dental Surgery, Royal College of Surgeons, formerly President Odontological Society of Great Britain.

Thoroughly revised and adapted to the use of American students and practitioners, by Thomas C. Stellwagen, M.A., M. D., D D.S., Professor of Physiology at the Philadelphia Dental College..

This old, well known manual of dental surgery to the British practitioner, has been greatly enlarged and improved in its revision for the American edition. The notes and illustrations added by Professor Stellwagen serve to give it a value it would not otherwise have had, bringing the newer and most important facts and discoveries of the last few years into notice. The manual, without revision, shows well how widely the dental practice of England differs from that of America, and hence the necessity for the descriptions "of modes of treatment demanded by the peculiarities of climate," as well as "the latest and most approved systems of operating." The chapter on artificial crowns is well and concisely written, and will be of interest to many whose only information on this subject has been obtained by "word of mouth," or from the experiences of writers in the different dental journals.

The typography and general appearance of this volume, in its new dress, is good.

